# The Predictive Values of Advanced Non-Small Cell Lung Cancer Patients Harboring Uncommon *EGFR* Mutations—The Mutation Patterns, Use of Different Generations of *EGFR*-TKIs, and Concurrent Genetic Alterations

**DOI:** 10.3389/fonc.2021.646577

**Published:** 2021-08-26

**Authors:** Jiarong Tan, Chengping Hu, Pengbo Deng, Rongjun Wan, Liming Cao, Min Li, Huaping Yang, Qihua Gu, Jian An, Juan Jiang

**Affiliations:** Department of Respiratory Medicine, Xiangya Hospital of Central South University, Changsha, China

**Keywords:** concurrent genetic alterations, tyrosine kinase inhibitors, uncommon *EGFR* mutations, EGFR, non-small cell lung cancer

## Abstract

**Introduction:**

Epidermal growth factor receptor (*EGFR*) *19del* and *L858R* mutation are known as “common mutations” in non-small cell lung cancer (NSCLC) and predict sensitivities to *EGFR* tyrosine kinase inhibitors (TKIs), whereas *20ins* and *T790M* mutations confer drug-resistance to *EGFR*-TKIs. The role of the remaining uncommon *EGFR* mutations remains elusive.

**Methods:**

We retrospectively screened a group of NSCLC patients with uncommon *EGFR* mutations other than *20ins* and *T790M*. The mutation patterns, use of different generations of *EGFR*-TKIs, and concurrent genetic alterations were analyzed. Meanwhile, a cohort of patients with single *19del* or *L858R* were included for comparison.

**Results:**

A total of 180/1,300 (13.8%) patients were identified. There were 102 patients with advanced or recurrent NSCLC that received first-line therapy of gefitinib/erlotinib/icotinib and afatinib and were eligible for analysis. The therapeutic outcomes among patients with common mutations (*EGFR*cm, n = 97), uncommon mutation plus common mutations (*EGFR*um+*EGFR*cm, n = 52), complex uncommon mutations (complex *EGFR*um, n = 22), and single uncommon mutations (single *EGFR*um, n = 28) were significantly different (ORRs: 76.3%, 61.5%, 54.5%, and 50.0%, respectively, p = 0.023; and mPFS: 13.3, 14.7, 8.1, and 6.0 months, respectively, p = 0.004). Afatinib showed superior efficacy over gefitinib/erlotinib/icotinib in *EGFR*cm (ORR: 81.0% *vs*. 75.0%, p = 0.773; mPFS: 19.1 *vs*. 12.0m, p = 0.036), *EGFR*um+*EGFR*cm (ORR: 100% *vs*. 54.5%, p = 0.017; mPFS: NE *vs*. 13.6m, p = 0.032), and single *EGFR*um (ORR: 78.6% *vs*. 21.4%, p = 0.007; mPFS: 10.1 *vs*. 3.0m, p = 0.025) groups. Comprehensive genomic profiling by Next Generation Sequencing encompassing multiple cancer-related genes was performed on 51/102 patients; the mPFS of patients without co-mutation (n = 16) and with co-mutations of tumor-suppressor genes (n = 31) and driver oncogenes (n = 4) were 31.1, 9.2, and 12.4 months, respectively (p = 0.046). *TP53* mutation was the most common co-alteration and showed significantly shorter mPFS than *TP53* wild-type patients (7.0 *vs*. 31.1m, p < 0.001). Multivariate analysis revealed that concurrent *19del/L858R* and tumor-suppressor gene alterations independently predicted better and worse prognosis in patients with uncommon mutations, respectively.

**Conclusions:**

Uncommon *EGFR* mutations constitute a highly heterogeneous subgroup of NSCLC that confer different sensitivities to *EGFR*-TKIs with regard to the mutation patterns. Afatinib may be a better choice for most uncommon *EGFR* mutations. Concurrent *19del/L858R* and tumor-suppressor gene alterations, especially *TP53*, can be established as prognostic biomarkers.

## Introduction

Epidermal growth factor receptor (*EGFR*) mutation is the most common oncogenic alteration in NSCLC, occurring in about 50% of Asian (1) and 10–15% of Caucasian patients (2). Exon 19 deletions and exon *21 L858R* substitutions are known as “common mutations” as they account for approximately 85–90% of all *EGFR* mutations and predict responses to *EGFR*-TKIs (3). *EGFR* mutations other than *19del* and *L858R* are known as “uncommon mutations”, which can occur alone or coexisted with other *EGFR* mutations (termed “complex mutations”) (4, 5). These uncommon mutations constitute a highly heterogeneous group with varied responses to *EGFR*-TKIs which have not been fully elucidated. The presence of drug-resistant mutations including exon 20 insertions (*20ins*) and *T790M* mutation usually showed poor responses to both first- and second-generation *EGFR*-TKIs (6, 7). As for uncommon *EGFR* mutations other than *20ins* and *T790M*, *G719X*, *L861Q*, and *S768I* are frequently observed and showed sensitivities to *EGFR*-TKIs, with ORR and mPFS of 41.6% and 7.7 months, though not as favorable as common mutations (8). Nevertheless, evidence on the clinical responses of other uncommon *EGFR* mutations remains elusive.

Prospective data regarding the activity of *EGFR*-TKIs against uncommon *EGFR* mutations are limited because only a few randomized clinical trials of *EGFR*-TKIs involved patients with uncommon mutations (9–12). Researchers analyzed the clinical data from the NEJ002 study involving 10 participants harboring uncommon mutations, and the results showed that gefitinib was ineffective against *G719X* or *L861Q* mutation (13). In contrast, several retrospective studies observed moderate efficacy of first-generation *EGFR*-TKIs against uncommon mutations, with ORR ranging from 13.27% to 48.40%, and mPFS ranging from 5.0 to 7.7 months (8, 14, 15). Meanwhile, increasing evidence has suggested improved efficacy of second-generation *EGFR*-TKIs on patients with uncommon mutations. A combined post-hoc analysis based on data from serial LUX-Lung trials reported high efficacy of afatinib against certain types of uncommon *EGFR* mutations, especially *G719X*, *L861Q*, and *S768I* (6). Based on these findings, afatinib was approved by the US Food and Drug Administration (FDA) in 2018 for patients with advanced NSCLC harboring these mutations. The widespread access of the highly sensitive Next Generation Sequencing (NGS) technology in clinical practice and the implementation of liquid-based mutation detection assays can identify an expanded spectrum of uncommon *EGFR* mutations of which the optimal treatment regimen is warranted further investigation.

The improvement of molecular detection technologies could identify both targetable driver mutations and some other concurrent alterations that may potentially be served as predictive markers for the efficacy of *EGFR*-TKIs treatment. An increasing number of studies reported that co-occurring abnormalities such as mutations in *TP53* and *RB1*, and amplification in *MET* and *ERBB2* were associated with significantly shorter PFS in patients with common *EGFR* mutations when they received targeted therapy (16, 17). However, it remains unclear whether these co-mutations would affect the clinical outcomes of patients harboring uncommon *EGFR* mutations.

In the present study, we investigated the therapeutic outcomes of NSCLC patients harboring uncommon *EGFR* mutations other than *20ins* and *T790M* who received first-line therapy of gefitinib/erlotinib/icotinib and afatinib. The study also evaluated a number of clinical variables as predictive or prognostic factors including mutation patterns, use of different generations of *EGFR*-TKIs, and additional concurrent genetic alterations.

## Materials and Methods

### Patients

**Figure 1:**
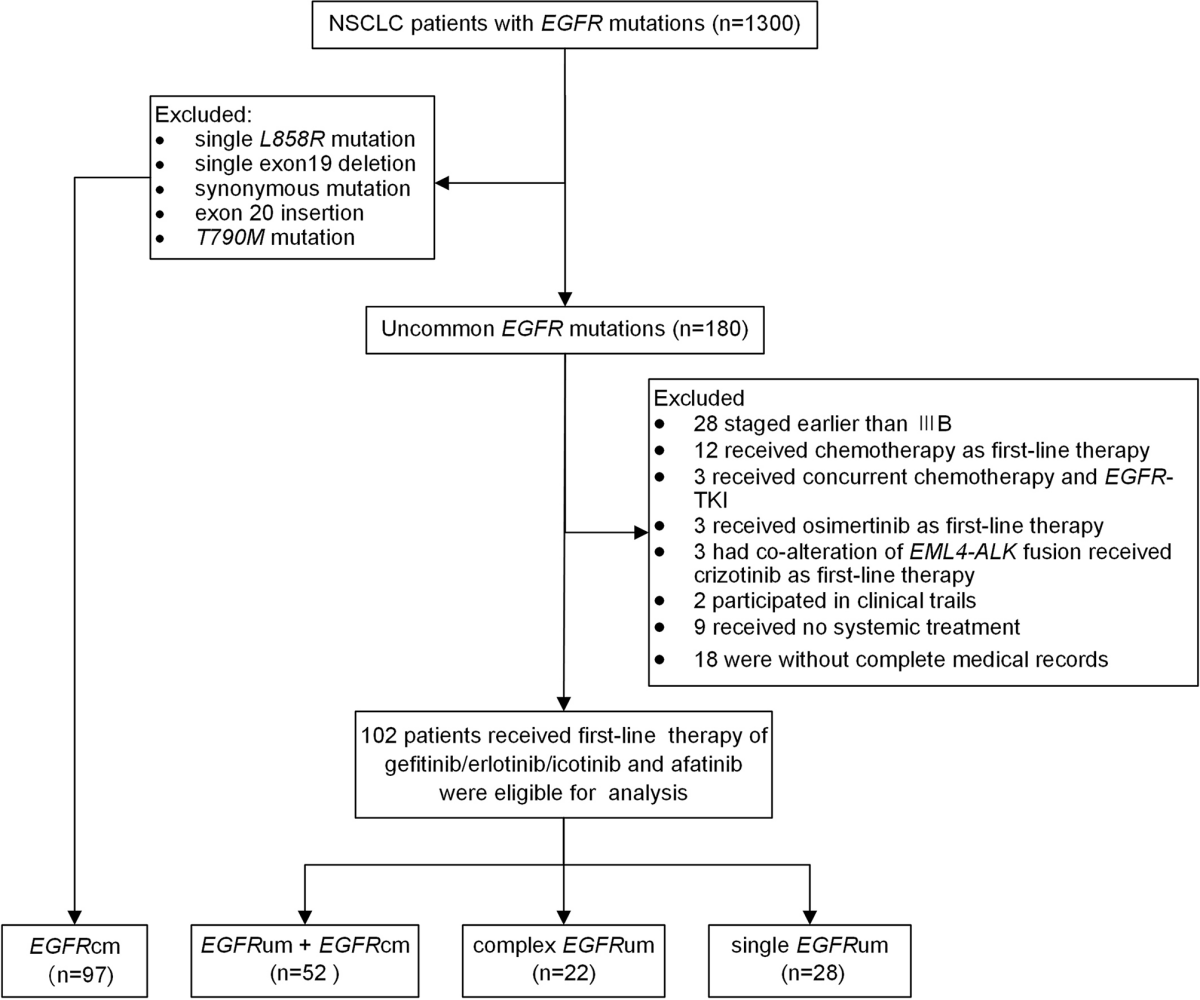
The flow chart for patient inclusion. NSCLC, non-small cell lung cancer; *EGFR*, epidermal growth factor receptor; TKI, tyrosine kinase inhibitor; *EGFR*cm, common *EGFR* mutations; *EGFR*um, uncommon *EGFR* mutations.

From February 2016 to June 2020, 1,300 patients with histologically or cytologically confirmed NSCLC who had *EGFR* mutations were retrospectively screened. One hundred eighty patients with uncommon *EGFR* mutations other than exon 20 insertions and *T790M* mutation were identified. Of which, 102 patients with advanced or recurrent disease who received gefitinib/erlotinib/icotinib or afatinib as first-line therapy were eligible for survival analysis (**Figure 1**). Besides, a cohort consisting of 97 NSCLC patients with single common *EGFR* mutations who received first-line *EGFR*-TKIs therapy during the same period were enrolled for comparison. The study was approved by the Medical Ethics Committee of Xiangya Hospital, Central South University (IRB (S) No.201907700).

### Clinical Data Collection and Efficacy Evaluation

Clinical data and therapeutic information were collected and analyzed, including age, sex, smoking status, Eastern Cooperative Oncology Group (ECOG) performance status, histologic type, *EGFR* mutation pattern, concurrent genetic alterations, *EGFR*-TKI use, and treatment outcomes. The disease stages were defined according to the eighth edition of the Lung Cancer Stage Classification System. *EGFR*-TKI treatment was initiated as per the physicians’ decision. Imaging examinations including chest Computed Tomography scanning (showing the liver and adrenal glands), brain Magnetic Resonance Imaging, and whole-body bone scan were performed every 8–12 weeks as a routine clinical procedure or as needed otherwise, to evaluate the treatment response and disease progression. The tumor response was assessed according to the Response Evaluation Criteria in Solid Tumours (RECIST; version 1.1) (18), including complete response (CR), partial response (PR), stable disease (SD), and progressive disease (PD). The objective response rate (ORR) was defined as the percentage of patients who achieved CR or PR. Progression-free survival (PFS) was measured as the period from the initiation of treatment to disease progression or death from any cause. Patients who had not experienced progression at the data cutoff date (January 15, 2021) or missing at the follow-up were censored.

### *EGFR* Mutation Testing

*EGFR* mutation status was assessed by amplification refractory mutation system (ARMS) or next generation sequencing (NGS). All samples were obtained prior to *EGFR*-TKIs treatment. ARMS was performed using tissue specimens, according to the protocol of the ADx-ARMS kit (Amoy Diagnostics, Xiamen, China), which is designed to identify a total of 29 *EGFR* mutations occurring within exons 18–21. For NGS assay, tumor tissues or plasma cell-free DNA (cfDNA) were available for targeted sequencing of genomic alterations using commercial gene panels **(** **Supplementary Tables 1**, **2** **)**. The detailed procedures and conditions followed previously established protocols (19, 20).

For 102 uncommon *EGFR*-mutant patients included, 83 (81.4%) were detected by NGS. Of which, 67 were profiled with tumor tissue specimens and 16 with plasma samples. The remaining 19 (18.6%) patients were confirmed by ARMs assay. The corresponding detection methods and sample types for each patient with uncommon *EGFR* mutations were shown in **Supplementary Table 3**. For 97 common *EGFR* mutated cases, 89 (91.8%) were tested using NGS, and 8 (8.2%) were determined by ARMS.

### Statistical Analysis

The chi-square test was used to compare qualitative data, and data with an expected frequency of <5 were analyzed using Fisher’s exact test. Mann–Whitney U test was applied to analyze continuous variables. Survival curves were plotted by the Kaplan–Meier method, and differences of median PFS among the subgroups were analyzed using the log-rank test. Univariate and multivariate Cox proportional hazards regression was performed to evaluate independent prognostic factors associated with PFS. Two-sided P values < 0.05 were considered statistically significant. All analyses were performed using SPSS 26 (IBM SPSS Statistics. Inc., Chicago, IL, USA).

## Results

### Patients’ Characteristics

A total of 1,300 patients with NSCLC and *EGFR* mutations were screened. There were 180 (13.8%) patients identified with uncommon *EGFR* mutations other than *20ins* and *T790M*, including 79 (44%) patients with a coexisting common *EGFR* mutation (*19del* or *L858R*), 39 (22%) patients with complex uncommon *EGFR* mutations, and 62 (34%) patients with single uncommon *EGFR* mutations such as *G719X* (n = 9, 5%), *L861Q* (n = 16, 9%), *S768I* (n = 4, 2%), and other single uncommon mutations (n = 33, 18%) **(** **Supplementary Figure 1** **)**. Noteworthily, 60/180 (33%) patients detected using the NGS method were found to have uncommon *EGFR* mutations occurring outside the most common 18–21 exons of the tyrosine kinase domain. Among them, 20 cases carried single mutation and 40 cases had complex mutations. Of the 40 complex mutations, 35 patients were combined with a common *EGFR* mutation.

Among the 180 patients with uncommon *EGFR* mutations other than *20ins* and *T790M*, 102 patients with advanced or recurrent disease received first-line therapy of gefitinib/erlotinib/icotinib and afatinib and were eligible for analysis. **Figure 1** shows the flow chart of patient inclusion. The median age at the initiation of *EGFR*-TKIs was 60 years old. The majority of the patients were female (55.9%), never smokers (66.7%), diagnosed with lung adenocarcinoma (92.2%), with ECOG performance status scored 0–1 (74.5%), and without brain metastases (70.4%). A total of 97 patients with single *19del* (50/97, 51.5%) or *L858R* (47/97, 48.5%) were included as common *EGFR* mutations (*EGFR*cm) for comparison. A significant difference in the use of first-line *EGFR*-TKIs was observed between the common mutations group and uncommon mutations group. No significant differences were observed for other baseline characteristics (**Supplementary Table 4**).

According to mutation patterns, patients with uncommon *EGFR* mutations were further grouped as follows: uncommon mutation plus common mutations (*EGFR*um+*EGFR*cm, n = 52), complex uncommon mutations (complex *EGFR*um, n = 22), and single uncommon mutations (single *EGFR*um, n = 28). There were 67 (65.7%) patients who received the first-generation TKIs (gefitinib/erlotinib/icotinib) and 35 (34.3%) patients who received the second-generation TKI (afatinib). Significant different *EGFR* mutation patterns were observed between the two generations of TKI cohorts, as there were more *EGFR*um+*EGFR*cm patients receiving gefitinib/erlotinib/icotinib but more complex *EGFR*um patients receiving afatinib (**Supplementary Table 5**). In the *EGFR*cm group, all baseline characteristics were comparable between the two treatment cohorts (**Supplementary Table 5**).

### Therapeutic Outcomes Among Patients With Different *EGFR* Mutation Patterns

At the time of data cutoff (January 15, 2021), the median follow-up was 26.0 months. The ORRs in patient subgroups of *EGFR*cm, *EGFR*um+*EGFR*cm, complex *EGFR*um, and single *EGFR*um were 76.3%, 61.5%, 54.5%, and 50.0%, respectively (p = 0.023). There was no significant difference in ORRs between *EGFR*cm and *EGFR*um+*EGFR*cm groups. The *EGFR*cm group had a significantly higher ORR than complex *EGFR*um (76.3% *vs*. 54.5%, p = 0.040) and single *EGFR*um groups (76.3% *vs*. 50.0%, p = 0.007).

In the gefitinib/erlotinib/icotinib cohort, the therapeutic responses remained significantly distinct among the four mutation groups, with ORRs of 75.0% in *EGFR*cm, 54.5% in *EGFR*um+*EGFR*cm, 44.4% in complex *EGFR*um, and 21.4% in single *EGFR*um, respectively (p < 0.001). Further analysis showed that the ORR of *EGFR*cm group was significantly higher than that of *EGFR*um+*EGFR*cm (75.0% *vs*. 54.5%, p = 0.021) and single *EGFR*um (75.0% *vs*. 21.4%, p<0.001) groups. Meanwhile, the ORR of *EGFR*um+*EGFR*cm group was also significantly higher than that of single *EGFR*um group (54.5% *vs*. 21.4%, p = 0.030). In the afatinib cohort, no significant differences in ORRs were observed among these groups. The clinical responses to *EGFR*-TKIs in patients with different mutation patterns were summarized in **Table 1**.

**Table 1:** Therapeutic responses among patients with different mutation patterns.

Comparison between groups	All patients		*EGFR*-TKIs^1st^		*EGFR*-TKI^2nd^	
	ORR (%)	p value	ORR (%)	p value	ORR (%)	p value
*EGFR*cm *vs*. *EGFR*um+*EGFR*cm	76.3 *vs*. 61.5	0.058	75.0 *vs* .54.5	0.021	81.8 *vs *.100	0.552
*EGFR*cm *vs*. complex *EGFR*cm	76.3 *vs*. 54.5	0.040	75.0 *vs *.44.4	0.109	81.8 *vs *.61.5	0.254
*EGFR*cm *vs*. single *EGFR*cm	76.3 *vs*. 50.0	0.007	75.0 *vs *.21.4	<0.001	81.8 *vs*. 78.6	1.000
*EGFR*um+*EGFR*cm *vs*. complex *EGFR*um	61.5 *vs*. 54.5	0.575	54.5 *vs*. 44.4	0.719	100 *vs*. 61.5	0.111
*EGFR*um+*EGFR*cm *vs*. single *EGFR*um	61.5 *vs*. 50.0	0.319	54.5 *vs*. 21.4	0.030	100 *vs*. 78.6	0.273
complex *EGFR*um *vs*. single *EGFR*um	54.5 *vs*. 50.0	0.749	44.4 *vs*. 21.4	0.363	61.5 *vs*. 78.6	0.420

EGFR, epidermal growth factor receptor; TKI, tyrosine kinase inhibitor; ORR, objective response rate; EGFRcm, common EGFR mutations; EGFRum, uncommon EGFR mutations.

**Figure 2:**
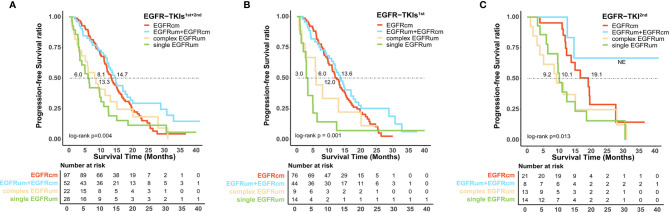
Patients harboring uncommon *EGFR* mutations with different mutation patterns exhibited diverse survival outcomes to the first-line therapy of *EGFR*-TKIs. Kaplan–Meier curves for progression-free survival in patients with different mutation patterns **(A)** in the first and second generations of *EGFR*-TKIs cohort; **(B)** in the first generation *EGFR*-TKIs cohort; and **(C)** in the second generation *EGFR*-TKI cohort. *EGFR*cm, common *EGFR* mutations; *EGFR*um, uncommon *EGFR* mutations.

For survival analysis, the mPFS in the *EGFR*cm, *EGFR*um+*EGFR*cm, complex *EGFR*um, and single *EGFR*um groups were 13.3 (95% CI 11.1–15.4), 14.7 (95% CI 12.5–16.8), 8.1 (95% CI 4.1–12.0), and 6.0 (95% CI 4.2–7.8) months, respectively (P = 0.004; **Figure 2A**). Furthermore, no significant difference in mPFS was found between the *EGFR*cm and *EGFR*um+*EGFR*cm groups. The mPFS of patients in the *EGFR*um+*EGFR*cm group was significantly longer than those in the complex *EGFR*um (14.7 *vs*. 8.1m, HR 1.924, 95% CI 1.105–3.351, p = 0.021) and single *EGFR*um (14.7 *vs*. 6.0m, HR 2.335, 95% CI 1.402–3.888, p = 0.001) groups.

**Figure 3:**
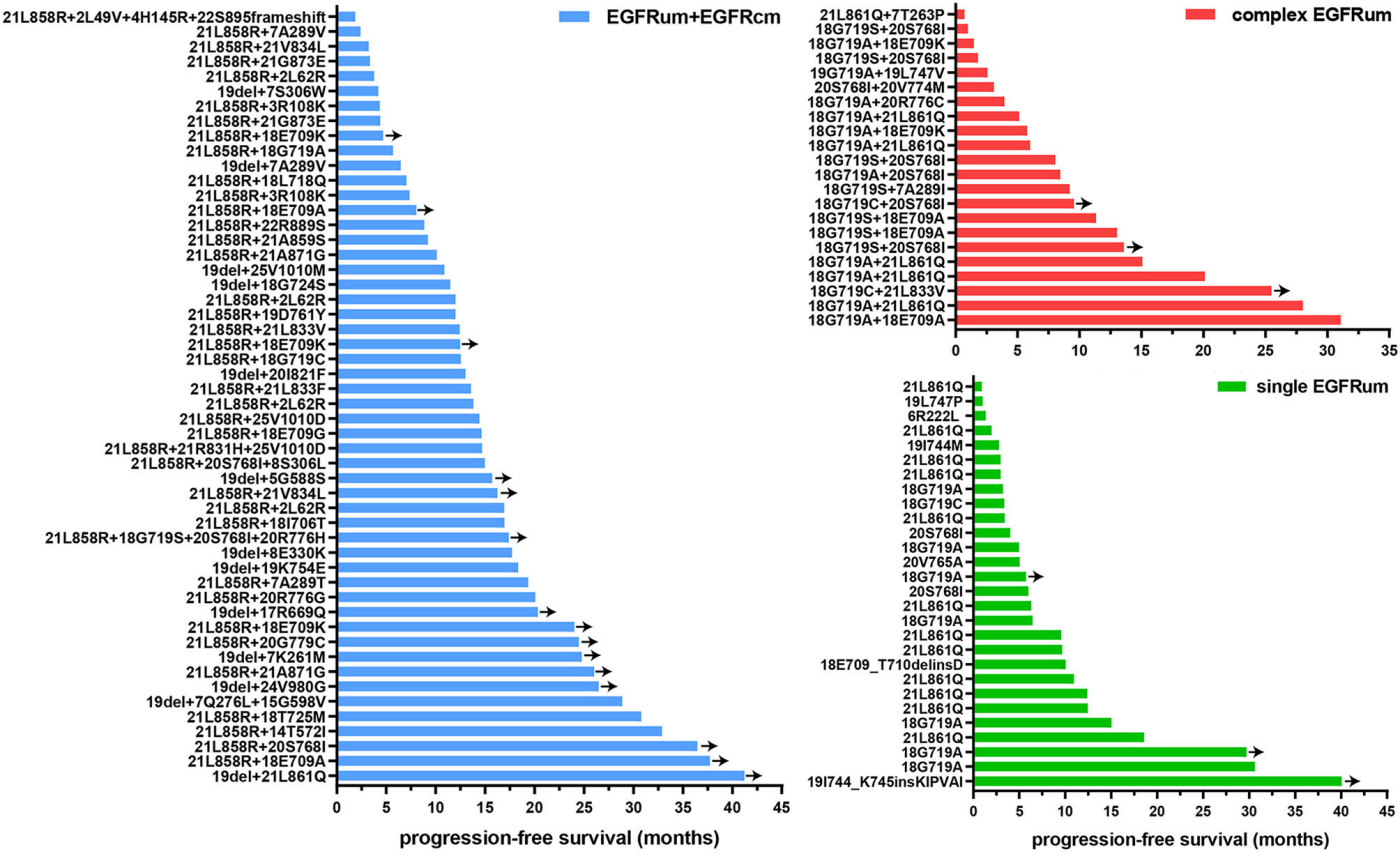
The progression-free survival (PFS) time for patients with specific mutation patterns received *EGFR*-TKIs as first-line therapy. Y-axis denotes mutation pattern, and x-axis indicates PFS. *EGFR*cm, common *EGFR* mutations; *EGFR*um, uncommon *EGFR* mutations.

We further performed a separate analysis in patients treated with different generations of *EGFR*-TKIs; the PFS curves of the four mutation groups in the two generations of TKI cohorts were shown in **Figures 2B, C**, respectively. In the gefitinib/erlotinib/icotinib cohort, the mPFS in *EGFR*cm and *EGFR*um+*EGFR*cm groups were comparable, while the mPFS of the *EGFR*um+*EGFR*cm group was significantly longer than that of the single *EGFR*um group (13.6 *vs*. 3.0m, HR 3.400, 95% CI 1.771–6.527, p < 0.001). In the afatinib cohort, although the mPFS in *EGFR*um+*EGFR*cm group was not yet reached, it remained statistically insignificant compared with the mPFS of *EGFR*cm group and prominently longer than those of complex *EGFR*um (NE *vs*. 9.2m, HR 6.397, 95% CI 1.411–28.995, p = 0.011) and single *EGFR*um (NE *vs*. 10.1m, HR 6.036, 95% CI 1.332–27.364, p = 0.012) groups. **Table 2** demonstrates the comparison of mPFS in patients with different mutation patterns. In addition, the PFS time for each specific mutation pattern was displayed in **Figure 3**.

**Table 2:** Median progression-free survival (mPFS) among patients with different mutation patterns.

Comparison between groups	All patients		*EGFR*-TKIs^1st^		*EGFR*-TKI^2nd^	
	mPFS (months)	p value	mPFS (months)	p value	mPFS (months)	p value
*EGFR*cm *vs*. *EGFR*um+*EGFR*cm	13.3 *vs*. 14.7	0.119	12.0 *vs*. 13.6	0.143	19.1 *vs*. NE	0.173
*EGFR*um+*EGFR*cm *vs*. complex *EGFR*um	14.7 *vs*. 8.1	0.021	13.6 *vs*. 6.0	0.115	NE *vs*. 9.2	0.011
*EGFR*um+*EGFR*cm *vs*. single *EGFR*um	14.7 *vs*. 6.0	0.001	13.6 *vs*. 3.0	<0.001	NE *vs*. 10.1	0.012
complex *EGFR*um *vs*. single *EGFR*um	8.1 *vs*. 6.0	0.616	6.0 *vs*. 3.0	0.243	9.2 *vs*. 10.1	0.923

EGFR, epidermal growth factor receptor; TKI, tyrosine kinase inhibitor; NE, not evaluable; EGFRcm, common EGFR mutations; EGFRum, uncommon EGFR mutations.

There were 24/102 patients that had uncommon mutations occurring beyond the exon 18–21 tyrosine kinase domain. Of them, 21 individuals had a coexisting *19del*/*L858R* and were classified into the *EGFR*um+*EGFR*cm group for analysis. Comparing the survival outcomes of the 21 patients to those who had single common mutations, the mPFS were 13.8 (95% CI 8.5–19.1) and 13.3 (95% CI 11.1–15.4) months, showing no statistically significant difference.

### Clinical Efficacy Between First-Line Therapy of Gefitinib/Erlotinib/Icotinib and Afatinib

**Figure 4:**
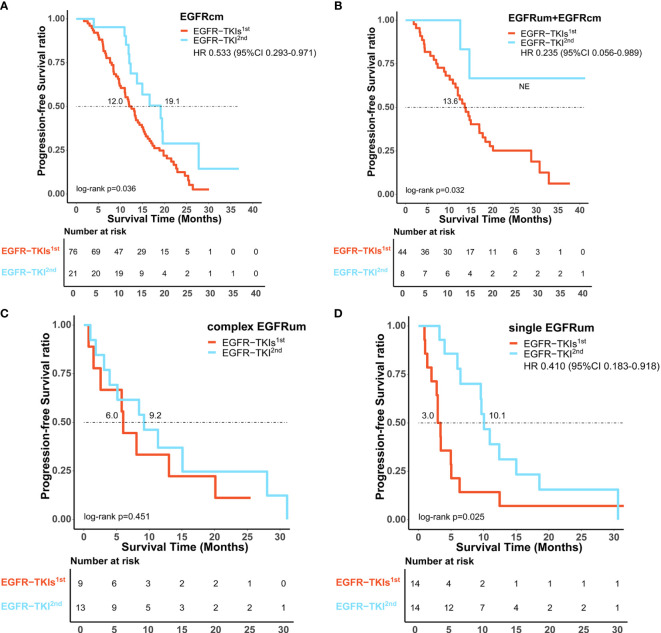
Comparison of survival outcomes between first-line therapy of gefitinib/erlotinib/icotinib (*EGFR*-TKIs^1st^) and afatinib (*EGFR*-TKI^2nd^). Afatinib showed significantly improved progression-free survival benefit over gefitinib/erlotinib/icotinib in patients subtypes of *EGFR*cm **(A)**, *EGFR*um+*EGFR*cm **(B)**, and single *EGFR*um **(D)**, but comparable median progression-free survival in the complex *EGFR*um group **(C)**. *EGFR*cm, common *EGFR* mutations; *EGFR*um, uncommon *EGFR* mutations; HR, hazard ratio; 95% CI, 95% confidence interval.

Comparing the first- and second-generation *EGFR*-TKIs, patients with common *EGFR* mutations receiving afatinib exhibited a comparable ORR (81.0% *vs*. 75.0%, p = 0.773) but significantly longer mPFS (19.1 *vs*. 12.0m, HR 0.533, 95% CI 0.293–0.971, p = 0.036, **Figure 4A**) than those receiving gefitinib/erlotinib/icotinib. For patients with uncommon *EGFR* mutations, the ORR of afatinib therapy was significantly higher than that of gefitinib/erlotinib/icotinib (77.1% *vs*. 46.3%, p = 0.003), but there was no significant difference in mPFS between the two treatment cohorts (12.4 *vs*. 10.9m, p = 0.333). In subgroup analysis of uncommon *EGFR* mutations, afatinib was associated with significantly favorable ORRs and mPFS than gefitinib/erlotinib/icotinib in patient subgroups of *EGFR*um+*EGFR*cm (100% *vs*. 54.5%, p = 0.017; NE *vs*. 13.6m, HR 0.235, 95% CI 0.056–0.989, p = 0.032, **Figure 4B**) and single *EGFR*um (78.6% *vs*. 21.4%, p = 0.007; 10.1 *vs*. 3.0m, HR 0.410, 95% CI 0.183–0.918, p = 0.025, **Figure 4D**). In the complex *EGFR*um group, no significant differences in ORRs (61.5% *vs*. 44.4%, p = 0.666) and mPFS were found between the two generations of TKIs (9.2 *vs*. 6.0m, p = 0.451, **Figure 4C**). The clinical efficacy between first-line therapy of gefitinib/erlotinib/icotinib and afatinib was shown in **Table 3**.

**Table 3:** Clinical efficacy between first-line therapy of gefitinib/erlotinib/icotinib and afatinib in patients with different *EGFR* mutation patterns.

Mutation patterns	ORR			mPFS (months), 95% CI		
	TKIs^1st^	TKI^2nd^	p value	TKIs^1st^	TKI^2nd^	P value
***EGFR*cm**	75.0%	81.0%	0.773	12.0 (9.7–14.3)	19.1 (11.8–26.5)	0.036
***EGFR*um**	46.3%	77.1%	0.003	10.9 (6.4–15.4)	12.4 (9.2–15.6)	0.333
*EGFR*um+*EGFR*cm	54.5%	100.0%	0.017	13.6 (10.7–16.4)	NE	0.032
Complex *EGFR*um	44.4%	61.5%	0.666	6.0 (5.3–6.7)	9.2 (2.4–16.0)	0.451
Single *EGFR*um	21.4%	78.6%	0.007	3.0 (2.2–3.7)	10.1 (8.5–11.7)	0.025

EGFR, epidermal growth factor receptor; TKI, tyrosine kinase inhibitor; ORR, objective response rate; PFS, progression-free survival; NE, not evaluable; EGFRcm, common EGFR mutations; EGFRum, uncommon EGFR mutations.

### The Predictive Value of Concurrent Genetic Alterations in Patients With Advanced NSCLC Harboring Uncommon *EGFR* Mutations

**Figure 5:**
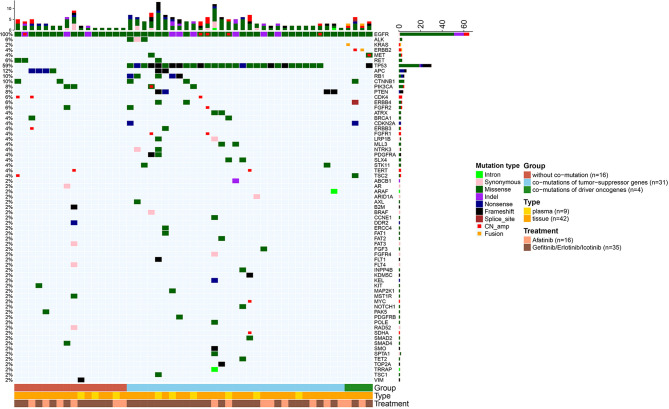
Heatmaps of genetic alterations of the 51 uncommon *EGFR* mutated patients who received NGS for testing multiple cancer-related genes. Patients were stratified into three subgroups according to the identified concurrent genetic alterations.

**Figure 6:**
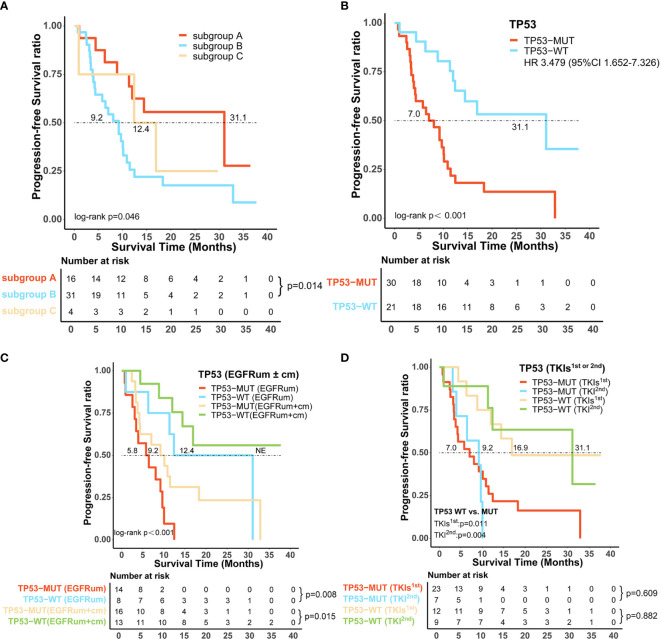
Certain co-alterations were associated with inferior survival outcomes in patients with uncommon *EGFR* mutations treated with *EGFR*-TKIs. **(A)** Progression-free survival (PFS) among patients without co-mutation (subgroup A), with co-mutations of tumor-suppressor genes (subgroup B) and driver oncogenes (subgroup C). **(B)** *TP53*-mutated (*TP53*-MUT) patients showed significantly shorter median PFS than *TP53* wild-type (*TP53*-WT) patients. The predictive value of *TP53* co-mutations in the survival outcomes of patients harboring uncommon *EGFR* mutations** (C)** with or without a combined common *EGFR* mutation, **(D)** receiving different generations of *EGFR*-TKIs. *EGFR*cm, common *EGFR* mutations; *EGFR*um, uncommon *EGFR* mutations; HR, hazard ratio; 95% CI, 95% confidence interval.

In addition to *EGFR*, information on concurrent genetic alterations was available in 51 patients with uncommon *EGFR* mutations who were identified by NGS using large sequencing gene panels. Genomic aberrations identified in these patients were indicated in **Figure 5**. There were 62.7% (32/51) of patients that had co-mutations of tumor-suppressor genes, including *TP53* (30/51, 59%), *RB1* (5/51, 10%), and *PTEN* (4/51, 8%). Co-mutations of driver oncogenes were found in 4/51 (8%) patients, including 1 patient with *MET* amplification, 2 with *ERBB2* amplification, and 1 with *KRAS* amplification. *ALK*, *ROS1*, *BRAF*, and *RET* alterations were not found because of the limited sample size. According to identified co-mutations, the patients were divided into three subgroups including subgroup A (n = 16) without co-mutation, subgroup B (n = 31) with co-mutations of tumor-suppressor genes (*TP53*, *RB1*, *PTEN*), and subgroup C (n = 4) with co-mutations of driver oncogenes, irrespective of tumor-suppressor gene alterations (*MET*, *ERBB2*, *KRAS*). Corresponding mPFS in these subgroups were 31.1 months (95% CI 6.8–55.3), 9.2 months (95% CI 5.6–12.8), and 12.4 months (95% CI 0.0–28.1), respectively (p = 0.046; **Figure 6A**). The mPFS of subgroup B was significantly shorter than that of subgroup A (HR 2.657, 95% CI 1.187-5.949, p = 0.014), while no significant difference of mPFS was observed in subgroup C compared with subgroups A and B.

*TP53* was the most frequently identified co-occurring genomic alteration; we further investigated the role of *TP53* mutation in patients with uncommon *EGFR* mutations and found that *TP53* mutated (*TP53*-MUT) patients had a significantly shorter mPFS than those of *TP53* wild-type (*TP53*-WT) (mPFS:7.0 *vs*. 31.1m, HR 3.479, 95% CI 1.652–7.326, p < 0.001, **Figure 6B**). Subsequently, we stratified the 51 patients with uncommon *EGFR* mutations based on whether they had a combined common *EGFR* mutation; the results showed that the *TP53*-MUT group was associated with significantly worse mPFS regardless of the presence of common *EGFR* mutation (*EGFR*um+*EGFR*cm: 9.2m *vs*. NE, HR 3.378, 95% CI 1.194–9.556, p = 0.015; *EGFR*um only: 5.8 *vs*. 12.4m, HR 4.594, 95% CI 1.39–15.22, p = 0.008, **Figure 6C**). Likewise, patients with concomitant *TP53* mutation demonstrated significantly shorter mPFS than those with *TP53* wild-type both on gefitinib/erlotinib/icotinib (7.0 *vs*. 16.9m, HR 3.113, 95% CI 1.238–7.825, p = 0.011, **Figure 6D**) and afatinib therapy (9.2 *vs*. 31.1m, HR:13.685, 95% CI 1.532–122.215, p = 0.004, **Figure 6D**). Furthermore, for *TP53* co-altered patients, no significant difference in mPFS was found between the two generations of *EGFR*-TKIs regimens (7.0 *vs*. 9.2m, p = 0.609, **Figure 6D**).

### Prognostic Factors for the Clinical Outcomes of Patients Harboring Uncommon EGFR Mutations

The Cox regression model included variables such as age, sex, smoking status, histological types, Eastern Cooperative Oncology Group (ECOG) performance status, brain metastasis at the initiation of therapy, *EGFR*-TKI treatment, mutation patterns, and co-mutations. Univariate analysis was performed to screen variables for multivariate analysis. In 102 patients with uncommon *EGFR* mutations, multivariate analysis indicated that patients with a concomitant common *EGFR* mutation (*19del*/*L858R*) were associated with longer PFS (p = 0.003, HR 0.500, 95% CI: 0.315–0.792), while patients with non-adenocarcinoma and poor ECOG PS score were associated with shorter PFS (p = 0.004, HR 3.221, 95% CI 1.447–7.168; and p = 0.005, HR 2.111, 95% CI 1.258–3.541, respectively) (**Table 4**). In 51 patients who had uncommon *EGFR* mutations with comprehensive tumor genomic information, the multivariate analysis additionally identified that co-mutations of tumor-suppressor genes were also independently associated with poorer PFS (p = 0.001, HR 3.545, 95% CI 1.632–7.703) (**Supplementary Table 6**).

**Table 4:** Cox regression analysis for prognostic factors in the 102 advanced NSCLC patients harboring uncommon *EGFR* mutations who received *EGFR*-TKIs as first-line therapy.

Variables	Univariate analysis			Multivariate analysis		
	HR	95% CI	P value	HR	95% CI	P value
Age ≥60 years old	0.996	0.643–1.543	0.987			
Female sex	0.795	0.514–1.232	0.305			
Never-smoker	0.895	0.567–1.413	0.634			
Non-adenocarcinoma	3.869	1.783–8.396	0.001	3.221	1.447–7.168	0.004
ECOG PS: 2–4	4.978	1.064–2.918	0.028	2.111	1.258–3.541	0.005
Brain metastasis at the initiation of therapy	0.963	0.592–1.565	0.877			
Afatinib therapy	0.792	0.493–1.272	0.335			
Uncommon mutations with a combined *19del*/*L858R*	0.490	0.315–0.762	0.002	0.500	0.315–0.792	0.003

HR, hazard ratio; 95% CI, 95% confidence interval; ECOG PS, Eastern Cooperative Oncology Group performance status.

## Discussion

In this study, we enrolled a group of NSCLC patients with uncommon *EGFR* mutations and performed a comprehensive analysis focusing on the mutation patterns, use of different generations of *EGFR*-TKIs, and concurrent genetic alterations. Our results suggested that uncommon *EGFR*-mutant NSCLC can be further stratified into various mutation subgroups which exhibit distinct therapeutic responses and survival outcomes to *EGFR*-TKIs. The second-generation TKI afatinib showed improved therapeutic effects than the first-generation TKI on patient’s subtypes of common mutations, uncommon mutation plus common mutations, and single uncommon mutations. Co-occurring tumor-suppressor gene alterations, especially *TP53*, are associated with poor survival outcomes in patients with uncommon *EGFR* mutations treated with *EGFR*-TKIs. To the best of our knowledge, this is the first study to explore the predictive value of concurrent genetic alterations focusing on uncommon *EGFR-*mutant NSCLC populations.

Patients with uncommon *EGFR* mutations showed variable ORRs and PFS among different mutation patterns. In the study by Keam et al., patients with coexisting uncommon mutations and *19del*/*L858R* showed comparable sensitivities to *EGFR*-TKIs with single common mutations (ORR of 68.8% and mPFS of 8.1 months) which were higher than patients harboring uncommon mutations without concomitant *19del*/*L858R* (ORR of 25% and mPFS of 1.4 months) (21). Herein, we observed 44% of patients had uncommon mutations coexisting with *19del*/*L858R*. In consistency with previous studies (5, 22), these patients showed similar therapeutic outcomes with patients harboring common mutations only and better outcomes than patients harboring single or complex uncommon mutations. In the complex uncommon mutations group, ORR was 54.5% and mPFS was 8.1 months, which was close to the results of Zhang et al. with an ORR of 71.0% and mPFS of 9.6 months (4). These findings suggested that complex uncommon mutations may also be effective targets for *EGFR*-TKI therapy. As for single uncommon mutations, earlier published data showed that patients of this subtype treated with first-generation TKIs had a significantly shorter mPFS than those of complex uncommon mutations (6.5 *vs*. 11.9 months, p = 0.010) (8). In the current study, however, no significant difference in mPFS was found between the two mutation subtypes, and larger datasets are required to validate this observation.

Owing to the advantage of the intact exon coverage of *EGFR* in NGS assays and the increase of sequencing depth, an increasing number of uncommon *EGFR* variants of unknown significance have been detected. In this study, 60 patients with uncommon *EGFR* mutations occurring beyond the exon 18–21 tyrosine kinase domain were identified. The biological function of these mutations and their sensitivities to *EGFR*-TKIs remain unclear while considering the large proportion; we did not exclude these patients. It should be noted, however, that most of these patients (21/24, 87.5%) met the inclusion criteria harboring a combined *19del/L858R* and were classified into the *EGFR*um+*EGFR*cm group for analysis. The clinical outcomes of the 21 patients showed no statistically significant difference compared with those who had *19del/L858R* alone. This indicates that the co-existence of uncommon *EGFR* mutations happening outside the exon 18–21 tyrosine kinase domain would not affect the sensitivities of common *EGFR* mutations to *EGFR*-TKIs.

Unlike the first-generation TKIs which reversibly bind to the ATP site of *EGFR*, afatinib is an oral pan-HER blocker that irreversibly binds to ErbB1 (*EGFR*), ErbB2 (HER2), and ErbB4, and inhibits signaling from all of these receptors (23). Therefore, the antitumor activity of afatinib should be more potent pharmacologically. The LUX-Lung 7 trial is the first prospective clinical trial to evaluate the clinical efficacy of afatinib and gefitinib as first-line therapy in patients with advanced NSCLC harboring common *EGFR* mutations. The results showed that afatinib demonstrated a statistically improved ORR and mPFS than gefitinib (ORR: 70.0% *vs*. 56.0%, p = 0.0083; mPFS:11.0 *vs*.10.9months, p = 0.017) (24). Consistently, we also observed superior efficacy in patients with common *EGFR* mutations using afatinib, with a significantly improved mPFS of 19.1 months. However, there have been no prospective studies compare these two generations of *EGFR*-TKIs in patients with uncommon mutations. Preclinical evidence suggests that afatinib has broad activity against uncommon *EGFR* mutations, with IC50 values much lower than those of first-generation *EGFR*-TKIs (25, 26). These *in vitro* preclinical data seem to be supported and reflected in the clinic. As shown by the results from a pooled analysis, afatinib demonstrated encouraging efficacy toward uncommon *EGFR* mutations, especially in patients whose tumors harbored major uncommon mutations (*G719X*, *L861Q*, and *S768I*, with or without any other mutation except *T790M* or an exon 20 insertion) and complex mutations, with ORR of 60.0% and 77.1%, respectively, and median time to treatment failure (TTF) of 10.8 months and 14.7 months, respectively (27). Shen et al. compared the effects of the two-generations of *EGFR*-TKIs and found that afatinib was more effective than gefitinib/erlotinib in the treatment of patients harboring uncommon *EGFR* mutations (mPFS:11.0 *vs*.3.6 months, p = 0.030), particularly for those lacking a combination of *19del* or *L858R* (mPFS:18.3 *vs*. 2.8 months, p = 0.070) (28). In a recently published retrospective study, afatinib as first-line therapy was reported to have a significantly greater therapeutic response than gefitinib or erlotinib (ORR:60.6% *vs*. 35.8%, p = 0.036) and a trend towards longer mPFS but not archived threshold of statistical significance (mPFS:8.8 *vs*. 12.0 months, p = 0.163) (29). Likewise, in the present study, for the whole group of uncommon *EGFR* mutations, afatinib achieved a significantly higher ORR but a comparable mPFS compared to gefitinib/erlotinib/icotinib. In subgroup analysis, afatinib was associated with significantly higher ORRs and longer mPFS in patient subgroups of uncommon mutation plus common mutations and single uncommon mutations. For complex uncommon mutations, these two treatment cohorts were similar. These observations indicated that afatinib could be a better choice in patients harboring common mutations and most uncommon mutations.

Previous reports suggested that concurrent genetic alterations within *EGFR* genomic aberrations play a role in the molecular resistance mechanisms to *EGFR*-TKI therapy, which might explain the shorter PFS in some patients (16, 17). The BENEFIT trial, for example, observed that patients with only common *EGFR* mutations had significantly longer mPFS than those possessing concurrent tumor-suppressor genes or other driver oncogene alterations (30). In the current study, we investigated the co-occurring genomic alterations in uncommon *EGFR*-mutant populations and explored their potential impact on the therapeutic outcomes of *EGFR*-TKIs. Similarly, our data suggested that patients carrying uncommon *EGFR* mutations with concurrent tumor-suppressor genes aberrations are associated with inferior outcomes on *EGFR*-TKIs therapy. As for patients harboring additional driver oncogene alterations, the mPFS was 12.39 months, which was comparable to the other two groups. However, this observation is not conclusive, due to the small number of cases. Further investigations with larger sample size are warranted to confirm our results.

We found that *TP53* mutation was the most frequently identified co-occurring genomic alteration in uncommon *EGFR*-mutant NSCLC, with an incidence rate of 59% (30/51), which is consistent with the reported 40–60% incidence of *TP53* co-mutations in *EGFR* mutation-positive patients in previous studies (17, 31, 32). The promoting effect of *TP53* mutations on early tumor progression of various malignancies, including NSCLC, has been confirmed by multiple reports (33, 34). In the present study, we noted that patients with advanced NSCLC carrying uncommon *EGFR* mutations with co-mutations of *TP53* were associated with a markedly shorter time to disease progression on initial *EGFR*-TKIs therapy. Further analysis showed that afatinib did not provide a survival benefit in patients with co-altered *TP53* compared with gefitinib/erlotinib/icotinib. However, the potential reasons that underlie the negative prognostic value of *TP53* mutations have not been well elucidated. It has been reported that the somatic mutations of *TP53* are related to the inactivation of P53 protein, which causes impaired tumor suppressor functions in anti-proliferation and apoptosis regulation, and is also associated with genomic instability and defects in DNA damage repair (35, 36). In addition, emerging evidence suggests that *TP53*-mutant lung cancers exhibited remarkably increased somatic mutation burden and higher expression of immune checkpoints such as PD-L1 (37). These findings indicate that *TP53* mutations may be somehow involved in the tumor adaptive immune escape, which might contribute to a favorable response toward immune checkpoint inhibitors but potential resistance to non-immunotherapy including molecular targeted therapy. Identification of additional co-mutations with predictive values using comprehensive tumor genomic profiling may help to tailor personalized therapeutic strategies to overcome primary resistance. However, there are currently no approved agents that specifically target *TP53* in NSCLC. Clinical trials assessing the efficacy of combination therapy are under investigation (38, 39), which may be a promising option for the treatment of *EGFR*-mutant NSCLC with co-alterations.

After adjustment for potential confounding factors, our results of multivariate analysis showed that concomitant common *EGFR* mutation is a predictor for better therapeutic outcomes in patients with uncommon *EGFR* mutations, which is consistent with the result from a prior study (29). Meanwhile, the presence of concurrent tumor-suppressor gene alterations is an independent risk factor for poor PFS. These observations further illustrated the predictive value of mutation patterns and certain concurrent alterations for patients with uncommon mutations.

### Limitations

There are some limitations in our study. Firstly, the single-center and retrospective design would involve potential biases. Secondly, although the majority of patients included in this study were tested using NGS assay, some uncommon *EGFR*-mutant variants beyond the 29 identifiable *EGFR* mutations may be missed for patients analyzed by ARMS. Thirdly, due to the limited number of cases in which multigene sequencing was performed, we failed to analyze more genetic co-alterations other than *TP53* mutations individually. Finally, we were unable to assess the overall survival time due to incomplete follow-up data after referral and the fact that a certain number of patients had not yet reached the endpoint events at the time of the data cutoff.

## Conclusion

The clinical outcomes of uncommon *EGFR* mutations are closely related to the mutation patterns, use of different generations of *EGFR*-TKIs, and concurrent genetic alterations. Patients carrying uncommon *EGFR* mutations coupled with *19del*/*L858R* are correlated with better efficacy than other mutation patterns. Afatinib provides improved therapeutic outcomes for most uncommon *EGFR* mutations and therefore could be a recommended option for these patients. The co-mutations of tumor-suppressor genes especially *TP53,* can serve as a predictive factor for poor prognosis.

## Data Availability Statement

The data presented in the study are deposited in the CNGB Sequence Archive (CNSA) of China National GeneBank DataBase (CNGBdb) repository (https://db.cngb.org/cnsa/), accession number CNP0001988.

## Ethics Statement

The studies involving human participants were reviewed and approved by The Medical Ethics Committee of Xiangya Hospital, Central South University (IRB (S) No.201907700). Written informed consent for participation was not required for this study in accordance with the national legislation and the institutional requirements.

## Author Contributions

JT participated in the study design, data collection and analysis, and drafted the manuscript. CH contributed to the study design, data acquisition, and manuscript review. PD contributed to study design, data interpretation, overall review, and funding acquisition. RW participated in data curation and visualization. LC, ML, HY, QG, JA, and JJ contributed to data acquisition. All authors contributed to the article and approved the submitted version.

## Funding

This work was supported by the National Natural Science Foundation of China (81502699,81600025); the National Key R&D Program of China (2016YFC1303300); the National Multidisciplinary Cooperative Diagnosis and Treatment Capacity Building Project for Major Diseases (Lung Cancer); and Xiangya clinical big data project of Central South University (Clinical big data project of lung cancer).

## Conflict of Interest

The authors declare that the research was conducted in the absence of any commercial or financial relationships that could be construed as a potential conflict of interest.

## Publisher’s Note

All claims expressed in this article are solely those of the authors and do not necessarily represent those of their affiliated organizations, or those of the publisher, the editors and the reviewers. Any product that may be evaluated in this article, or claim that may be made by its manufacturer, is not guaranteed or endorsed by the publisher.
