# Supplementary figures and images for: The Predictive Values of Advanced Non-Small Cell Lung Cancer Patients Harboring Uncommon EGFR Mutations—The Mutation Patterns, Use of Different Generations of EGFR-TKIs, and Concurrent Genetic Alterations
Source: Front Oncol. 2021 Aug 26;11:646577. doi: 10.3389/fonc.2021.646577 (PMC8426345; doi:10.3389/fonc.2021.646577)

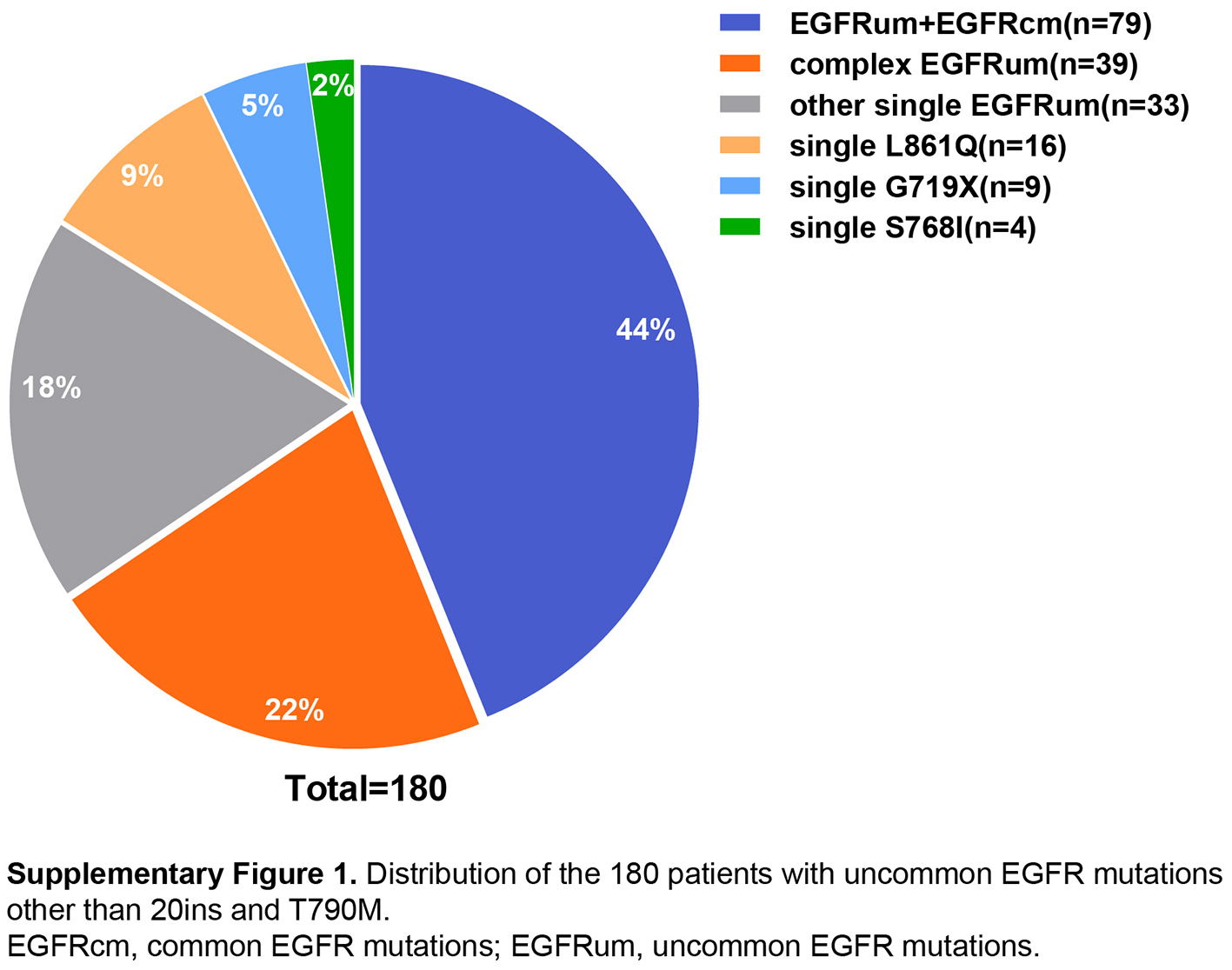

Supplement: Supplementary file 1 [file Image_1.jpeg]
